# *QuickStats:* Percentage* of Children and Adolescents Aged 5–17 Years Who Had Ever Received a Diagnosis of Attention-Deficit/Hyperactivity Disorder,^†^ by Urbanization Level^§^ and Age Group — National Health Interview Survey, United States, 2020–2022^¶^

**DOI:** 10.15585/mmwr.mm7305a6

**Published:** 2024-02-08

**Figure:**
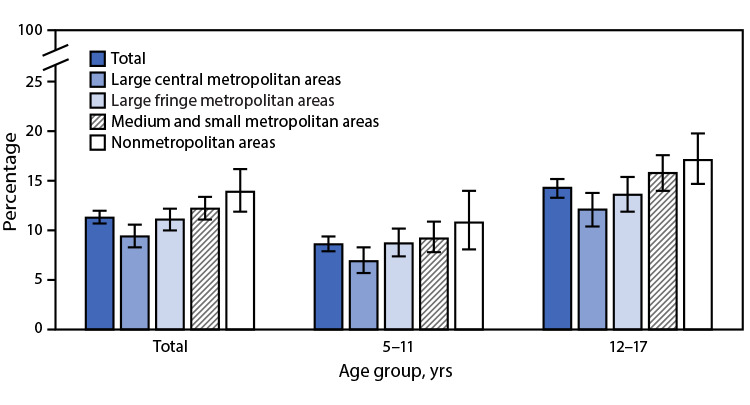
During 2020–2022, 11.3% of children and adolescents aged 5–17 years had ever received a diagnosis of ADHD. The percentage of children and adolescents who had ever received a diagnosis of ADHD increased with decreasing level of urbanization from 9.4% among those living in large central metropolitan areas to 13.9% among those living in nonmetropolitan areas. A similar pattern was seen among children aged 5–11 years (6.9% in large central metropolitan areas compared with 10.8% in nonmetropolitan areas) and children and adolescents aged 12–17 years (12.1% to 17.1%). Children and adolescents aged 12–17 years were more likely than were children aged 5–11 years to receive an ADHD diagnosis across all levels of urbanicity.

**Source:** National Center for Health Statistics, National Health Interview Survey, 2020–2022. https://www.cdc.gov/nchs/nhis.htm

